# Non-canonical Wnt signaling participates in Jagged1-induced osteo/odontogenic differentiation in human dental pulp stem cells

**DOI:** 10.1038/s41598-022-11596-9

**Published:** 2022-05-09

**Authors:** Chatvadee Kornsuthisopon, Ajjima Chansaenroj, Jeeranan Manokawinchoke, Kevin A. Tompkins, Nopadon Pirarat, Thanaphum Osathanon

**Affiliations:** 1 Dental Stem Cell Biology Research Unit, Faculty of Dentistry, Chulalongkorn University, 34 Henri-Dunant Rd. Pathumwan, Bangkok, 10330 Thailand; 2 Department of Pathology, Faculty of Veterinary Science, Chulalongkorn University, 39 Henri-Dunant Rd. Pathumwan, Bangkok, Bangkok, 10330 Thailand; 3 Office of Research Affairs, Faculty of Dentistry, Chulalongkorn University, Bangkok, 10330 Thailand; 4 Department of Anatomy, Faculty of Dentistry, Chulalongkorn University, Bangkok, 10330 Thailand

**Keywords:** Differentiation, Mesenchymal stem cells

## Abstract

Osteoblast differentiation requires the interaction of various cell signaling pathways to modulate cell responses. Notch and Wnt signaling are among the crucial pathways that control numerous biological processes, including osteo/odontogenic differentiation. The aim of the present study was to examine the involvement of Wnt signaling in the Jagged1-induced osteo/odontogenic differentiation in human dental pulp stem cells (hDPSCs). The Wnt-related gene expression was analyzed from publicly available data of Jagged1-treated human dental pulp cells. The mRNA expression of Wnt ligands (WNT2B, WNT5A, WNT5B, and WNT16) and Wnt inhibitors (DKK1, DKK2, and SOST) were confirmed using real-time polymerase chain reaction. Among the Wnt ligands, *WNT2B* and *WNT5A* mRNA levels were upregulated after Jagged1 treatment. In contrast, the Wnt inhibitors *DKK1*, *DKK2*, and *SOST* mRNA levels were downregulated. Recombinant WNT5A, but not WNT2B, significantly promoted in vitro mineral deposition by hDPSCs. Wnt signaling inhibition using IWP-2, but not DKK1, inhibited Jagged1-induced alkaline phosphatase (ALP) activity, mineralization, and osteo/odontogenic marker gene expression in hDPSCs. In conclusion, Jagged1 promoted hDPSC osteo/odontogenic differentiation by modulating the non-canonical Wnt pathway.

## Introduction

The Wnt pathway has crucial functions in various processes underlying the development and maintenance of tissue homeostasis^1^. Wnt signal cascades are divided into the canonical and non-canonical pathways. Canonical Wnt signal transduction occurs via β-catenin accumulation in the cytosol that subsequently translocates into the nucleus, modulating target gene expression^2^. The non-canonical Wnt pathway comprises the calcium-dependent pathway and planar cell polarity cascades, which do not require β-catenin accumulation^3^. Wnt/β‐catenin signaling activation enhanced the osteogenic differentiation of human dental pulp stem cell (hDPSCs)^4^, apical papilla stem cells^5^, and human periodontal ligament stem cells (hPDLSCs)^6–8^. These findings suggested that Wnt signaling was involved in the osteogenic differentiation of dental-related cells.

Notch signaling is involved in many cell responses during development and homeostasis^9^. Direct binding between a Notch ligand and receptor leads to receptor cleavage, releasing the Notch intracellular domain (NICD). This domain then translocates into the nucleus, forms a complex with other transcription factors, and activates Notch target gene transcription^10^. Previous studies demonstrated that a Notch ligand, Jagged1, potentiated osteo/odontogenic differentiation in various cells isolated from dental tissues, including human dental pulp cells, stem cells isolated from human exfoliated deciduous teeth, and hPDLSCs^11,12^. Therefore, Notch signaling has been demonstrated to play an important role in osteo/odontogenic differentiation.

Cross-regulation between the Notch and Wnt pathways through their components has been reported in previous studies^13–15^. Glycogen synthase kinase 3 beta (GSK-3β), a component of the Wnt/β-catenin signaling cascade, is involved in the temporal switch between Notch and Wnt signaling in mouse myogenesis^13^. GSK-3β modulated Notch signaling by directly phosphorylating NICD^14^. NICD activity stimulated the effect of Lymphoid enhancer-binding factor 1 (LEF‐1), a transcription factor involved in the canonical Wnt/β-catenin signaling, specific promoters^15^. However, the crosstalk that occurs between these two pathways to regulate hDPSC osteogenic differentiation remains unresolved. The aim of the present study was to investigate the participation of Wnt signaling in Jagged-induced osteo/odontogenic differentiation in hDPSCs.

## Results

### Stem cell characterization

**Figure 1:**
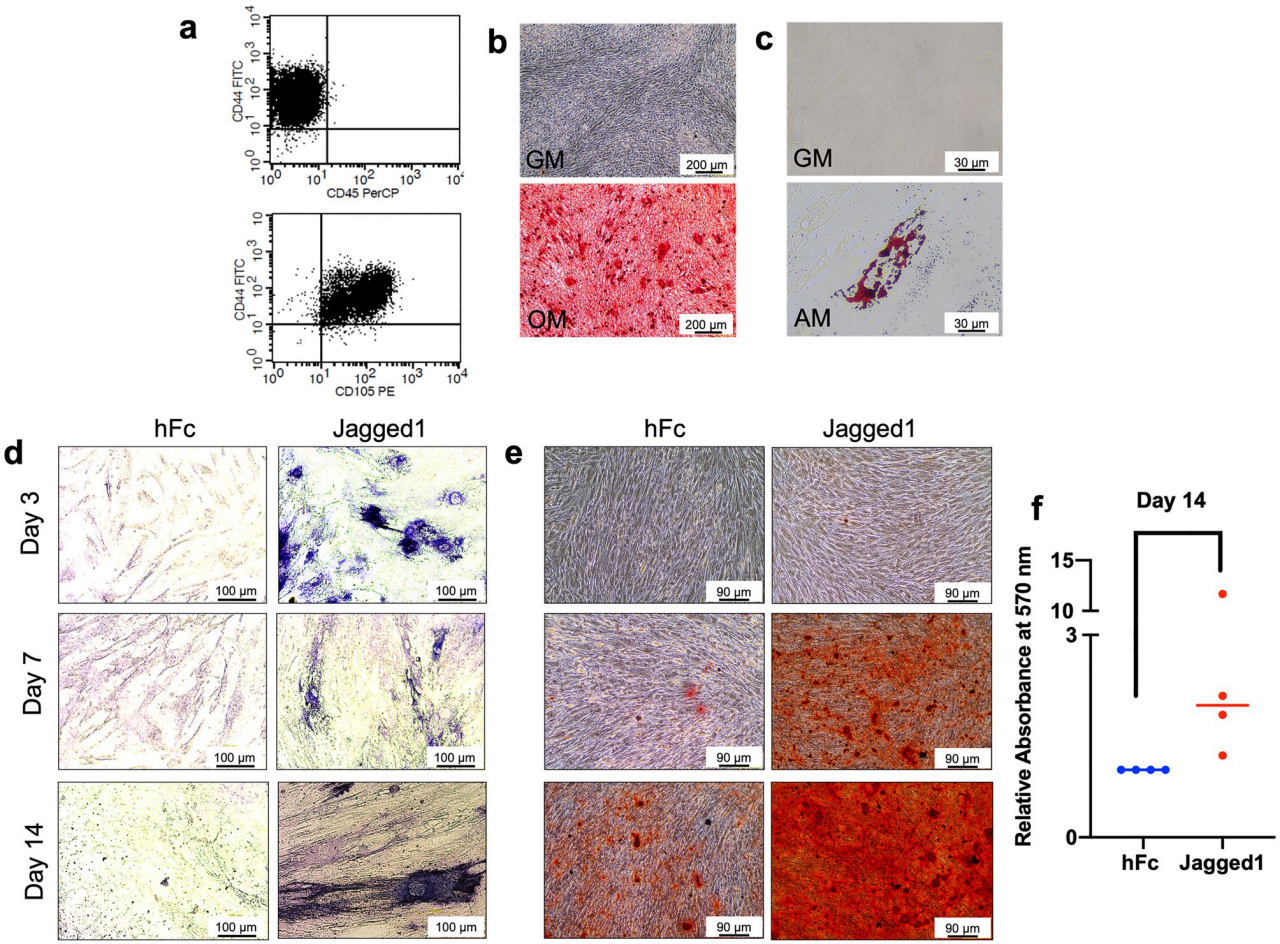
Indirect immobilized Jagged1 promotes hDPSC osteo/odontogenic differentiation. Mesenchymal stem cell surface markers were examined using (**a**) flow cytometry. Multilineage differentiation towards the osteo/odontogenic and adipogenic lineages was examined using (**b**) alizarin red s staining and (**c**) oil red o staining, respectively. Cells were seeded on Jagged1 immobilized surface and maintained in osteo/odontogenic medium. Cells on hFc-immobilized surfaces were used as the control. The cells were cultured in osteo/odontogenic medium for 3, 7, and 14 days. Osteo/odontogenic differentiation was evaluated by (**d**) ALP staining and (**e**) in vitro mineral deposition using alizarin red s staining. The alizarin red s staining was solubilized, and (**f**) the absorbance was measured at 570 nm. Bars indicate a significant difference between groups (*p* < 0.05).

The isolated cells exhibited a spindle-shaped fibroblast-like morphology. Surface protein analysis revealed the expression of mesenchymal stem cell (MSC)-related surface markers (CD44 and CD105) and the absence of the hematopoietic cell marker CD45 (Fig. 1a). To determine the cells’ multilineage differentiation potential, osteo/odontogenic and adipogenic induction were performed. Increased mineral deposition was observed compared with the undifferentiated control after osteo/odontogenic induction for 14 days (Fig. 1b). The cells maintained in adipogenic medium exhibited an increased intracellular lipid accumulation compared with the cells cultured in normal growth medium after induction for 16 days (Fig. 1c).

### Indirect immobilized Jagged1 promoted osteo/odontogenic differentiation in hDPSCs

hDPSCs were seeded on indirect immobilized Jagged1 tissue culture surfaces and maintained in osteo/odontogenic medium for 3, 7, and 14 days. Cells on human IgG Fc fragment (hFc) immobilized surfaces were used as the control. Increased ALP staining and mineralization were observed at all time points in the Jagged1-treated group (Fig. 1d and e). Analyzed quantitatively, significantly enhanced mineral deposition was observed in the Jagged-treated cells (*p* = 0.028) (Fig. 1f). These results confirmed our previous results that Notch signaling activation using immobilized Jagged1 promoted the osteo/odontogenic differentiation of hDPSCs in vitro.

### Notch regulated numerous Wnt-related genes

**Figure 2:**
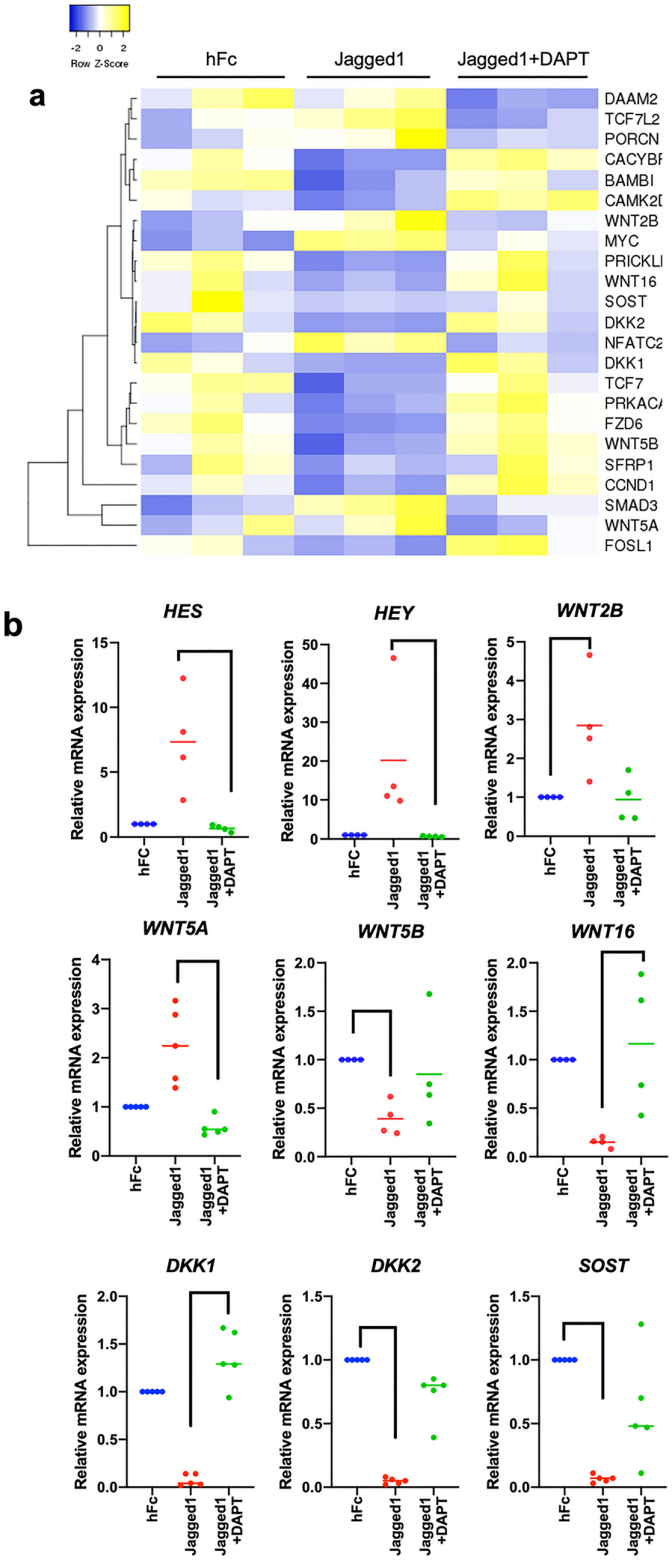
Notch regulates Wnt-related gene expression. (**a**) Heatmap demonstrating the significantly differential expression of Wnt-related genes in the Jagged1-treated condition. (**b**) The differential gene expression of selected genes in Jagged1-treated hDPSCs was confirmed using real-time polymerase chain reaction. Bars indicate a significant difference between groups (*p* < 0.05).

To determine the expression of Wnt related genes in the cells treated with Jagged1, bioinformatic analysis was performed. The significant differential gene expression is illustrated as a Heatmap (Fig. 2a). The bioinformatic analysis of the gene expression profile of the Jagged1-treated hDPSCs revealed that Notch regulated numerous Wnt-related genes. Specific upregulated and downregulated genes (*WNT2B, WNT5A, WNT5B, WNT16, DKK1, DKK2*, and *SOST*) were selected to validate the RNA sequencing results using real-time quantitative polymerase chain reaction. Among the Wnt proteins, *WNT2B* and *WNT5A* mRNA levels were upregulated, whereas *WNT5B* and *WNT16* were downregulated after Jagged1 treatment. Furthermore, the canonical Wnt inhibitors *DKK1, DKK2*, and *SOST* exhibited decreased mRNA levels in the Jagged1-treated hDPSCs (Fig. 2b). In addition, pre-treatment with DAPT abolished the Jagged1-induced *HES1* and *HEY1* mRNA expression in hDPSCs, confirming that DAPT effectively inhibited Notch signaling (Fig. 2b). Correspondingly, DAPT pretreatment attenuated the effect of Jagged1 on Wnt-related gene expression.

**Figure 3:**
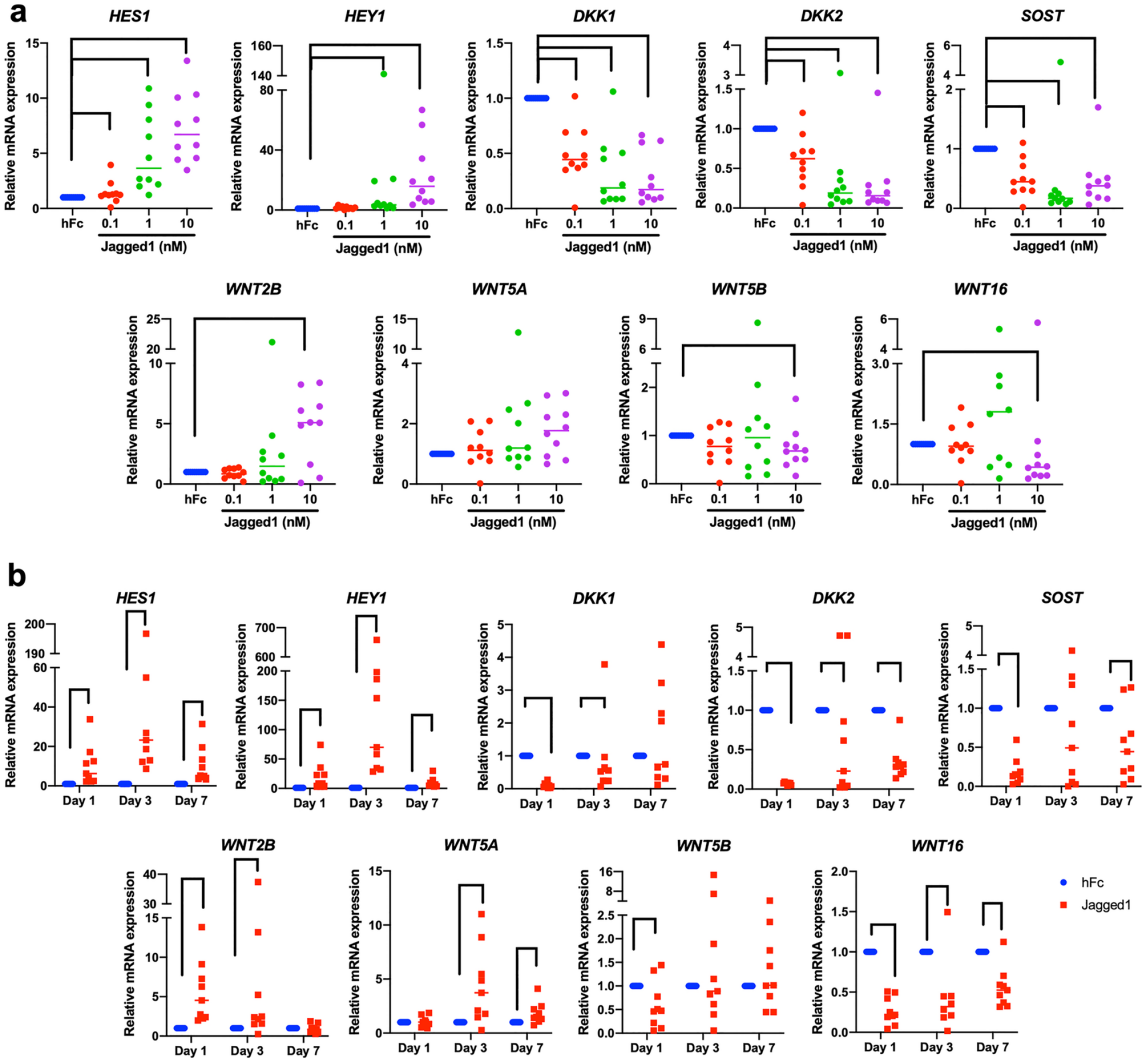
Dose and time course experiment of Jagged1 promotes Wnt related gene expression in hDPSCs. Correlation of Notch and Wnt pathways. (**a**) The mRNA levels of Notch-related genes (*HES1* and *HEY1*) and Wnt-related genes *(WNT2B, WNT5A, WNT5B, WNT16, DKK1, DKK2, and SOST)* were measured using real-time polymerase chain reaction after exposed to different Jagged1 concentrations for 24 h. (**b**) The mRNA expression of hDPSCs treated with 10 nM Jagged1 was evaluated at day 1, 3, and 7. Bars indicate a significant difference between groups (*p* < 0.05).

Jagged1 promoted Notch target gene, *HES1* and *HEY1,* expression in a dose-dependent manner (Fig. 3a). Similarly, the effect of Jagged1 on *DKK1*, *DKK2*, *SOST*, *WNT2B*, *WNT5A*, *WNT5B*, and *WNT16* expression was also dose-dependent. Jagged1 upregulated *HES1* and *HEY1* at day 1, 3, and 7 (Fig. 3b). Higher fold increase was noted at day 3. The mRNA expression of *DKK1, DKK2, SOST* and *WNT16* decreased at all time points. *WNT2B* mRNA levels were significantly increased at day 1 and 3 and *WNT5A* mRNA levels were significantly increased at day 3 and 7.

### WNT5A enhanced the osteo/odontogenic differentiation in hDPSCs

**Figure 4:**
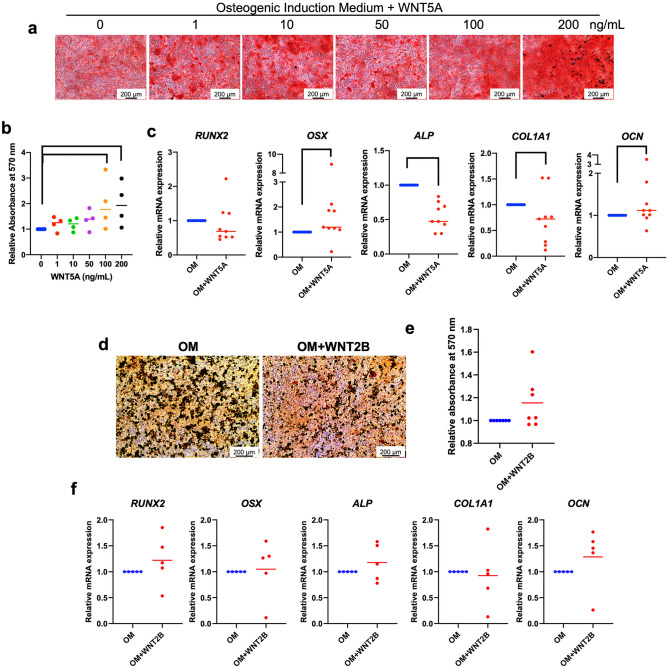
WNT5A enhances the osteo/odontogenic differentiation in hDPSCs. The cells were maintained in osteo/odontogenic medium with either (**a**–**c**) recombinant WNT5A or (**d**–**f**) WNT2B supplementation. Cells cultured in osteo/odontogenic medium were used as the control. (**a** and **d**) Mineral deposition was evaluated at day 14. (**b** and **e**) The solubilized staining was measured and quantitated. (**c** and **f**) The mRNA levels of osteo/odontogenic marker genes were measured using real-time polymerase chain reaction on day 7. Bars indicate a significant difference between groups (*p* < 0.05).

Because only two Wnt ligands, WNT2B and WNT5A, were upregulated in the Jagged1-treated condition, the effects of exogeneous WNT2B and WNT5A on osteo/odontogenic differentiation were evaluated. Cells were maintained in osteo/odontogenic medium supplemented with 1, 10, 50, 100, or 200 ng/ml recombinant WNT5A. Cells cultured in osteo/odontogenic medium were used as the control. WNT5A at concentrations of 100 and 200 ng/ml significantly enhanced mineral deposition at day 14 (*p* = 0.045, *p* = 0.028; respectively) (Fig. 4a and b). Therefore, hDPSCs were treated with 200 ng/ml WNT5A and subsequently evaluated for osteo/odontogenic marker gene expression at day 7. WNT5A significantly induced *OSX* and *OCN* mRNA expression (*p* = 0.045, *p* = 0.034; respectively); however, *ALP* and *COL1A1* mRNA levels were decreased compared with the control (Fig. 4c). No significant difference was observed in *RUNX2* mRNA levels. In contrast, when the cells were treated with WNT2B, no significant difference in mineral deposition or osteo/odontogenic marker gene expression was observed in the cells treated with 500 ng/ml WNT2B compared with the control (Fig. 4d–f). These results suggested that WNT5A promoted hDPSC osteo/odontogenic differentiation.

### DKK1 did not significantly inhibit Jagged1-induced osteo/odontogenic differentiation

**Figure 5:**
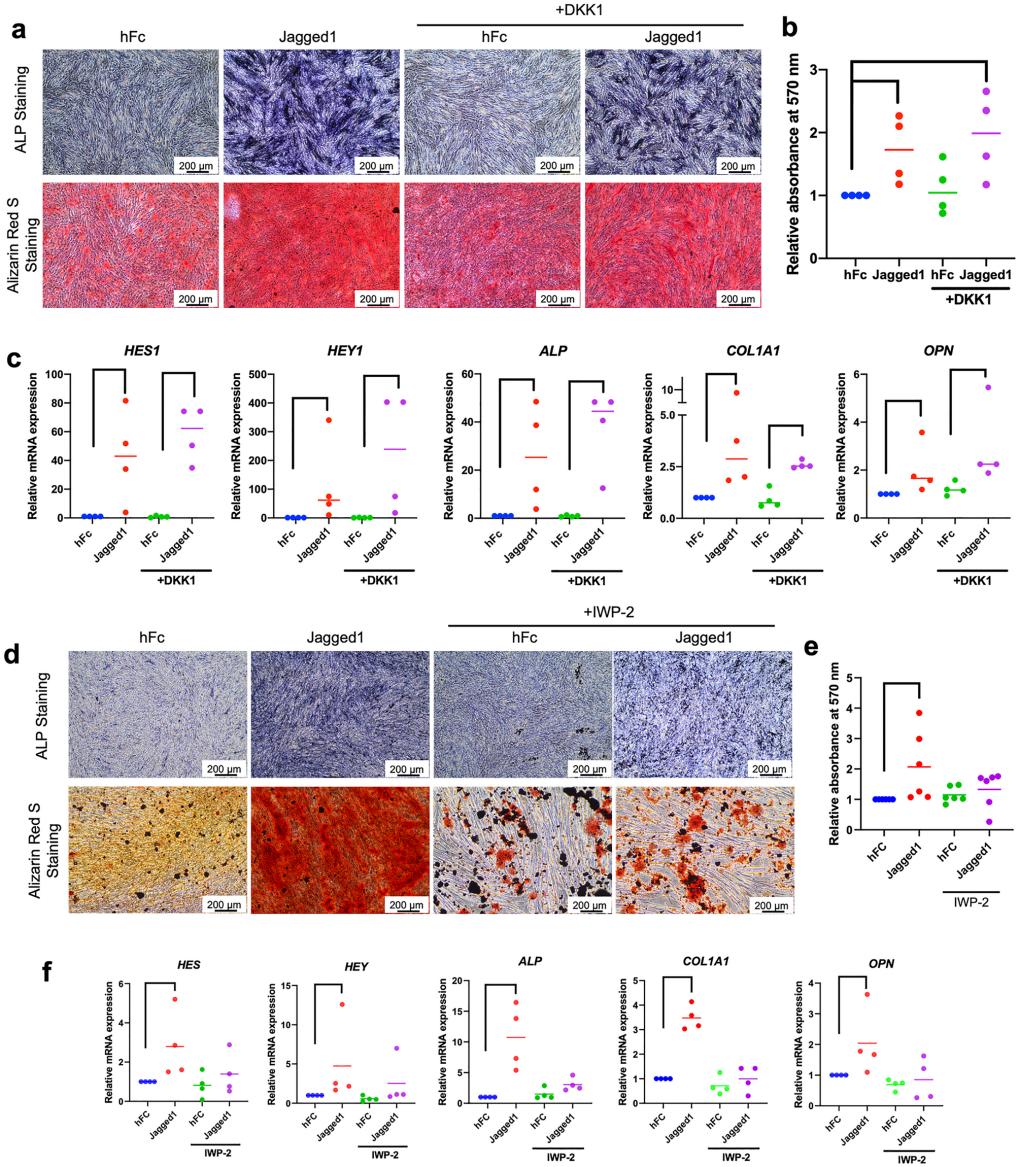
IWP-2 attenuates the osteo/odontogenic differentiation induced by Jagged1. Cells were pretreated with DKK1 or IWP-2 30 min prior to seeding on Jagged1 surfaces and further maintain in osteo/odontogenic induction medium. (**a** and **d**) ALP enzymatic activity and mineral deposition was examined at day 7 and 14, respectively. (**b** and **e**) The solubilized alizarin red s staining was measured and quantitated. (**c** and **f**) The mRNA levels of osteo/odontogenic marker genes were measured using real-time polymerase chain reaction. Bars indicate a significant difference between groups (*p* < 0.05).

The Wnt inhibitor DKK1 was employed to evaluate the influence of Wnt signaling on the Jagged1-induced osteo/odontogenic differentiation in hDPSCs. Cells were pretreated with 0.5 µg/ml DKK1 for 30 min prior to seeding on the Jagged1-immobilized surface and maintained in osteo/odontogenic medium. DKK1 pretreatment failed to inhibit the effects of Jagged1-induced ALP enzymatic activity, mineral deposition, and osteo/odontogenic marker gene expression (Fig. 5a–c).

### IWP–2 attenuated Jagged1-induced osteo/odontogenic differentiation in hDPSCs

IWP-2 inhibits the Wnt pathway by targeting Porcupine, an intracellular protein that interferes with Wnt protein secretion. The cells were pre-treated with 25 µM IWP-2 for 30 min prior to seeding on the Jagged1-immobilized surface. IWP-2 pretreatment resulted in decreased ALP enzymatic activity and mineral deposition after Notch activation during osteo/odontogenic differentiation at day 7 and 14 (Fig. 5d and e). IWP-2 abolished the effects of the Jagged1-induced *ALP*, *COL1A1*, and *OPN* mRNA expression at day 3 after osteo/odontogenic induction (Fig. 5f).

### Reciprocal control between Notch and Wnt pathway

**Figure 6:**
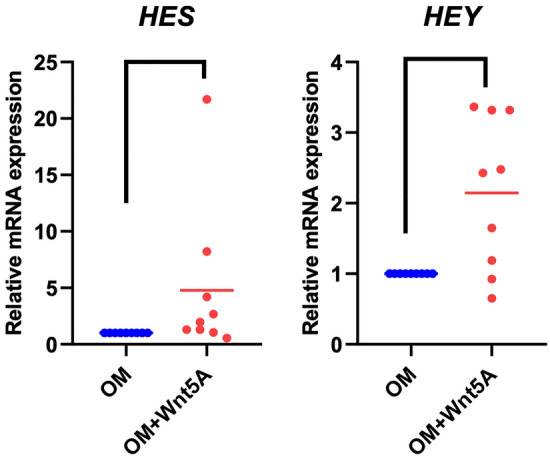
Reciprocal control between Notch and Wnt pathway. The cells were cultured in osteo/odontogenic medium supplemented with 200 ng/ml WNT5A for 7 days. The effects of WNT5A on Notch signaling were determined by the mRNA levels of Notch-related genes (*HES1* and *HEY1*). Bars indicate a significant difference between groups (*p* < 0.05).

To determine the interaction between Wnt and Notch, hDPSCs were treated with 200 ng/ml WNT5A and maintained in osteo/odontogenic medium for 7 days. The results revealed that WNT5A upregulated Notch target gene, *HES1* and *HEY1,* mRNA expression (*p* = 0.002, *p* = 0.036; respectively) (Fig. 6). These results suggested a feedback interaction between the Notch and Wnt pathways.

## Discussion

Osteoblast differentiation requires orchestrated crosstalk between cell signaling pathways^16,17^. Various signaling pathways, including transforming growth factor beta (TGF-β)/bone morphogenetic proteins (BMPs), Notch, Hedgehog, neural epidermal growth factor-like 1 protein (NELL-1), and Wnt/β-catenin, fibroblast growth factor (FGF), vascular endothelial growth factor (VEGF), and extracellular regulated-signal kinase (ERK) signaling, induce osteogenic lineage commitment and differentiation of MSCs^18,19^. All these pathways act in concert to regulate the osteo/odontogenic differentiation processes and ultimately bone formation. Among these key signaling pathways, Notch and Wnt signaling are highly conserved pathways that control several developmental processes, including bone formation and regeneration^20,21^. Notch exhibits inhibitory effects on osteogenic differentiation at the initial commitment stage, whereas it promotes terminal differentiation. The Wnt pathway modulates osteogenic differentiation via an interaction with the Runx2 promoter^18^. The previous publications by our group and other groups discussed the multi-communication crosstalk between Notch and other signaling pathways during osteogenic differentiation. Understanding the integration between signaling pathways provides insight into the molecular mechanisms that govern stem cell differentiation. The present study investigated the potential crosstalk between Notch and Wnt signaling in regulating hDPSC osteo/odontogenic differentiation. The isolated cells exhibited the characteristics that met the criteria for mesenchymal stem cells^22^. However, it should be noted that these cells were heterogeneous in nature. The responses of specific subpopulations require further analysis.

Our results revealed that Jagged1-mediated Notch activation upregulated several Wnt-related genes, including the canonical and non-canonical Wnt ligands WNT2B and WNT5A, respectively. Furthermore, WNT5A treatment promoted mineralization, suggesting that Wnt5A might participate in the Notch-induced osteo/odontogenic differentiation of hDPSCs. The inhibition experiments revealed that IWP-2, but not DKK1, attenuated the osteo/odontogenic differentiation of Jagged1-treated hDPSCs. These results imply the involvement of the non-canonical Wnt, WNT5A, in the Jagged1-induced mineralization in hDPSCs.

Among the Notch ligands, the effectiveness of Jagged1 on Notch activation and osteo/odontogenic induction was demonstrated in previous studies^11,23–25^. Protein kinase C delta (PKCδ) was previously reported to interact and phosphorylate the NICD, leading to NICD stability and increased nuclear translocation. Jagged1 treatment increased the binding between NICD and PKCδ, thereby promoting Notch activation^23^ In addition, Jagged1 attenuated transcription factor TWIST expression, which is a negative regulator of osteogenic differentiation^11,24,25^. Several Notch components were expressed in the tissue adjacent to the pulp capping area, which influenced the differentiation of pulp cells into odontoblast-like cells in response to pulp injury^26^. Therefore, the activation of Notch signaling at the specific sites is crucial to control odonto/osteogenic differentiation. These results demonstrated that Notch positively regulates osteoblast differentiation. Similarly, our results indicated that Jagged1-mediated Notch activation drove hDPSCs toward the osteo/odontogenic lineage as demonstrated by increased ALP staining and mineral deposition. However, the effect of Notch signaling on osteoblast differentiation remains unresolved. Notch signaling attenuated osteoblast differentiation in murine bone marrow stromal cells by repressing the transactivating ability of Runx2 through Hes/Hey protein activity^27^. Thus, the effect of Notch signaling on osteogenic differentiation depends on the cell type and is stage specific^28^.

In the present study, Wnt signaling-related genes were differentially expressed in hDPSCs after Jagged1-treatment. WNT2B and WNT5A were among the most highly upregulated genes. We observed that WNT5A promoted mineral deposition in hDPSCs, Additionally, the osteo/odontogenic -related genes were upregulated concomitantly with the downregulation of ALP during the mineralization stage of osteo/odontogenic differentiation^29^. Therefore, we hypothesized that this non-canonical Wnt might be involved in Notch-induced osteo/odontogenic differentiation. Similarly, WNT5A was previously reported to promote osteogenic differentiation of several cell types e.g., human bone marrow-derived mesenchymal stem cells^30^ and human adipose tissue-derived mesenchymal stromal cells^31^. Further, functionalized biomaterial-mediated activation of WNT5A enhanced the osteogenic lineage commitment of hMSCs in vitro and rat calvarial defect healing^32^. These results indicate the important role of WNT5A in osteogenesis. However, previous reports observed that WNT5A exerted an inhibitory effect on osteoblastic differentiation of an immortalized human periodontal ligament fibroblast cell line^33^. Similarly, WNT5A gene silencing promoted mineralization in human osteoblasts obtained from osteoarthritis patients^34^. We speculated that the different cell types and inflammatory condition might alter cell responses and lead to inconsistent findings.

To investigate whether the non-canonical Wnt pathway participated in Notch-induced hDPSC osteo/odontogenic differentiation, we inhibited the canonical Wnt pathway using DKK1. DKK1 antagonizes canonical Wnt signaling by targeting low density lipoprotein receptor-related protein 5/6, which is a component of the Wnt receptor complex responsible for mediating downstream canonical Wnt signaling^35^. In addition, we utilized IWP-2, which attenuates Wnt protein transportation and secretion. Hence, both canonical and non-canonical pathways are inhibited^36^. We found that the effect of Jagged1 was inhibited only when the cells were treated with IWP-2, but not DKK1, as demonstrated by the decreased mineral deposition along with downregulated osteo/odontogenic -related genes. In addition, the β-catenin accumulation and *Axin2* mRNA expression in Jagged1-treated hDPSCs had a similar profile compared with control, indicating that canonical Wnt signaling was not activated during Notch activation (Supplementary Fig. S1). In contrast, upregulation of Calcium/calmodulin-dependent protein kinase II (CaMKII) and Receptor tyrosine kinase‑like orphan receptor 2 (ROR2), components related to non-canonical Wnt pathway, was observed after Jagged1 treatment for 24 h. This effect was attenuated by DAPT, as demonstrated by the significantly decreased mRNA expression of these two genes in the cells that were pretreated with DAPT prior to being seeded on the Jagged1 surface (Supplementary Fig. S2). These results suggest that the non-canonical Wnt pathway might participate in the Jagged1-induced osteo/odontogenic differentiation in hDPSCs.

Crosstalk between Notch and Wnt was described in previous studies; however, in most studies, only canonical Wnt signaling was analyzed. Co-culture between MSC and hematopoietic stem cells (HSCs) were pivotal for HSC proliferation and maintenance^24,37^. MSCs expressed β-catenin, whose gene transactivation resulted in Jagged1 expression, which functioned as a paracrine ligand to activate Notch signaling in HSCs^38,39^. These results suggested that the regulation of hematopoiesis requires Notch-canonical Wnt interaction.

Crosstalk between Notch and Wnt signaling was also reported during bone formation and regeneration^40,41^. Notch signaling activation downregulated the expression of Wnt inhibitors (Sost and Dkk), thereby promoting bone formation by osteocytes^40,41^. Notch and canonical Wnt activation were demonstrated to promote bone regeneration and healing in a mouse cortical defect model^42^. In contrast, negative regulation between these signaling pathways has also been reported. Canonical Wnt promoted MSC differentiation towards the osteoblast lineage; however, Hes1-induced Notch activation attenuated this effect of canonical Wnt, thereby inhibiting MSC osteogenic differentiation^40,43^. Crosstalk between Notch and canonical Wnt was observed during the early stages of intramembranous bone regeneration^44^. Notch regulated osteoprogenitor cell proliferation, and this effect was terminated by canonical Wnt activation, leading to osteogenic differentiation^44^. Similarly, the NICD was reported to occupy and inhibit T-cell factor/lymphoid enhancer factor at its binding functional domain, suggesting the underlying mechanism by which Notch exerts an inhibitory effect on the canonical Wnt signaling pathway^45,46^.

The present study demonstrated the interaction between Notch and non-canonical Wnt signaling. Our results showed that Jagged1 and WNT5A treatment promoted mineralization in hDPSCs. A feedback regulation between these two pathways was observed, as demonstrated by an upregulation of Wnt5A expression by Jagged1 and increased Notch-related gene expression by WNT5A treatment. These findings imply that there might be a reciprocal control between Notch and non-canonical Wnt to regulate hDPSC osteo/odontogenic differentiation. A previous study reported that Wnt5A/serine-threonine Ca(2+)/calmodulin-dependent protein kinase II (CaMKII) signaling mediated Notch signaling activation by interacting with the silencing mediator of retinoic acid and thyroid hormone receptor (SMRT), which is a key co-repressor of Notch signaling. CaMKII phosphorylated SMRT, leading to SMRT translocation to the cytoplasm, where it was further degraded by the proteasome^47^. However, further investigation is required to prove this hypothesis. To the best of our knowledge, there has been no report on Notch-non canonical Wnt crosstalk regarding the osteo/odontogenic differentiation of hDPSCs. When differentiating into odontoblasts, DPSCs become polarized, which is a prerequisite and fundamental step for cell differentiation^48,49^. Non-canonical Wnt is a key signaling pathway that controls cell polarization through the non-canonical Wnt/planar cell polarity pathway^50^. Thus, we speculate that non-canonical Wnt signaling might participate in the Jagged1-induced osteo/odontogenic differentiation in hDPSCs by regulating cell polarization. However, whether this interaction occurs at the transcriptional level or via elements in the signal transduction chain remains unexplored. In-depth investigations are needed to further reveal the intracellular mechanism between these two signaling pathways.

In summary, the present study found that Notch and non-canonical Wnt signaling had crosstalk during hDPSC osteo/odontogenic differentiation. Notch activation stimulated hDPSC osteo/odontogenic differentiation and promoted WNT2B and WNT5A expression, which suggests the involvement of Wnt signaling in Notch-induced osteo/odontogenic differentiation. Moreover, DKK1 failed to abolish the effect of Notch signaling. However, IWP-2 inhibition of Wnt protein transportation and secretion attenuated the effect of Notch, suggesting that the non-canonical Wnt pathway might partially act downstream of Notch in the osteo/odontogenic differentiation of hDPSCs. Further investigation is required to prove this hypothesis by using specific knockout molecules related to non-canonical Wnt followed by Notch activation to elucidate the specific role of non-canonical Wnt signaling in the crosstalk with Notch in hDPSC osteo/odontogenic differentiation.

## Materials and methods

### Cell isolation and culture

The hDPSCs were harvested from extracted impacted third molars with informed consent at the Faculty of Dentistry, Chulalongkorn University. The protocol was approved by the Human Research Ethics Committee of Chulalongkorn University (approval no. 037/2021) and conducted in accordance with the guidelines of the Biosafety Committee at the Faculty of Dentistry, Chulalongkorn University. Briefly, dental pulp tissues were minced and placed on 35-mm tissue culture dishes for cell outgrowth. The growth medium was composed of Dulbecco’s Modified Eagle Medium (DMEM, cat. no. 11960, Gibco, USA) containing 10% fetal bovine serum (FBS, cat. no. 10270, Gibco, USA), 2 mM L-glutamine (GlutaMAX-1, cat. no. 35050, Gibco, USA), 100 unit/mL penicillin, 100 μg/mL streptomycin, and 250 ng/mL amphotericin B (Antibiotic–Antimycotic, cat. no. 15240, Gibco, USA). The cells were maintained at 37 °C in a humidified 5% carbon dioxide atmosphere. The culture medium was changed every 48 h. Subsequent assays were performed using cells between passages 3 and 7.

### Flow cytometry analysis

Surface protein expression was analyzed using flow cytometry. Cells were harvested and prepared as a single cell suspension. The cells were stained with fluorescence conjugated antibodies (1:50 dilution). The antibodies were FITC conjugated anti-human CD44 (Cat. No. 555478, BD Bioscience, USA), PE-conjugated anti-human CD105 (Cat. No. 21271054, Immuno Tools, Germany), and PerCP-conjugated anti-CD45 (Cat. No. 21810455, Abcam, USA). The mean fluorescence intensity was determined by a FACS^Calibur^ flow cytometer using CellQuest software (BD Bioscience, USA).

### Osteo/odontogenic differentiation

Cells were seeded on 24-well-plates (25,000 cells/well) and maintained in growth medium for 24 h. Subsequently, the medium was changed to osteo/odontogenic medium. The osteo/odontogenic medium consisted of growth medium supplemented with 50 µg/mL ascorbic acid (cat. no. A-4034, Sigma-Aldrich, St. Louis, MO, USA), 250 nM dexamethasone (cat. no. D8893, Sigma-Aldrich), and 5 mM β-glycerophosphate (cat. no. G9422, Sigma-Aldrich). In some experiments, the osteo/odontogenic medium was supplemented with either 1, 10, 50, 100, or 200 ng/ml recombinant WNT5A (R&D Systems Inc.) or 500 ng/ml WNT2b (R&D Systems Inc.) to activate Wnt signaling. In contrast, pre-treatment with Wnt inhibitors, 0.5 µg/ml DKK1 (R&D Systems Inc.), and 25 µM IWP-2 (Tocris Bioscience), was used to inhibit Wnt signaling.

### Adipogenic differentiation

Cells were seeded on 24-well-plates (12,500 cells/well) and maintained in growth medium for 24 h. Subsequently, the medium was changed to adipogenic medium, which was growth medium containing 0.1 mg/ml insulin (cat. no. 11070738 Sigma-Aldrich, USA), 1 μM dexamethasone (cat. no. D8893, Sigma-Aldrich, USA), 1 mM IBMX (cat. no. PHZ1124, Thermo Fisher Scientific, USA), and 0.2 mM indomethacin (cat. no. 53861, Sigma-Aldrich, USA). At 16 days, the intracellular lipid droplet accumulation was examined using oil red o staining.

### Jagged1 immobilization

Jagged1 was indirectly immobilized on the tissue culture surface as previously described^11^. Briefly, 24-well tissue culture plates were incubated with 50 μg/mL recombinant protein G (cat. no. 101201, Invitrogen, Rockford, IL, USA) for 16 h. Subsequently, the surface was incubated with 10 mg/mL bovine serum albumin solution (cat. no. A9418, Sigma-Aldrich) for 2 h and then incubated with 0.1, 1, or 10 nM rhJagged1/Fc recombinant protein (cat. no. 1277-JG, R&D Systems, Minneapolis, MN, USA) for 2 h. Sterile phosphate buffered saline (PBS) was used to wash the culture plates between each step. For the control condition, the human IgG Fc fragment (hFc, cat. no. 009000008, Jackson Immuno Research Labs, USA) was placed in the wells in amounts equal to the Jagged1 ligand.

To inhibit Notch signaling, the cells were pretreated with a γ-secretase inhibitor, 20 μM DAPT (cat. no. D5942, Sigma-Aldrich) for 30 min prior to being seeded on the Jagged1-immobilized surface.

### Bioinformatic analysis

The high throughput RNA sequencing raw read counts were downloaded from the NCBI Gene Expression Omnibus (series GSE94989)^12^. Wnt-related genes were listed according to the KEGG database (entry hsa04310)^51^. The RNA read counts of significantly different expressed genes were identified using NetworkAnalyst^52,53^ and an expression heatmap was generated using Heatmapper^54^.

### Polymerase chain reaction

Total cellular ribonucleic acid (RNA) was extracted using TRIzol reagent (RiboEx solution, cat. no. 301-001, GeneAll, Seoul, South Korea). The quality and quantity of the isolated RNA was examined using a Nanodrop instrument. One microgram of total cellular RNA was converted to cDNA using the ImProm-II Reverse Transcription System (cat. no. A3800, Promega, Madison, WI, USA). A CFX connect Real-Time PCR (Bio-Rad, Singapore) with FastStart Essential DNA Green Master (Roche Diagnostic, Mannheim, Germany) were used to perform the real-time quantitative polymerase chain reaction. The amplification profile was: 95 °C/20 s, 60 °C/20 s, and 72 °C/20 s for 45 cycles. Melt curve analysis was performed to determine product specificity. The mRNA levels of the target genes were normalized to the 18S gene, and the relative gene expression was quantified with the comparative Ct method (2^−ΔΔCt^ method)^55^. The oligonucleotide sequences of the primers are shown in Supplementary Table 1.

### Alkaline phosphatase staining

Cells were fixed with 4% paraformaldehyde solution for 10 min, followed by incubating in a 0.09% BCIP/NBT solution (BCIP/NBT tablets, Roche, USA) for 30 min at room temperature in the dark. After washing with deionized water, the ALP-stained cells were observed using an inverted light microscope.

### Alizarin red s staining

Cells were fixed with cold methanol for 10 min and gently washed with deionized water. The cells were then stained with a 2% alizarin red s solution (Sigma-Aldrich Chemical) for 3 min at room temperature with gentle agitation. Images of the stained cells were captured using an inverted light microscope. To indirectly measure the amount of calcium deposition, the staining was solubilized with 10% cetylpyridinium chloride monohydrate in 10 mM sodium phosphate at room temperature with gentle agitation for 15 min. The absorbance was measured at 570 nm with a microplate reader (Molecular Devices, Palo Alto, CA).

### Oil red O staining

Cells were fixed with 10% buffered formalin for 30 min, followed by incubating with a 0.2% Oil Red O solution for 15 min. Intracellular lipid accumulation was examined using an inverted light microscope (Olympus, USA).

### Immunofluorescence staining

Cells were fixed with 4% buffered formalin at room temperature for 10 min and permeabilized using 0.15% Triton-X100 in PBS. Horse serum (2% v/v) was used to inhibit a non-specific binding. Cells were stained with β-Catenin XP Rabbit mAb (cat. no. 8480, Cell Signaling, USA) at a 1:100 dilution at 4 °C overnight. Cells were incubated with biotinylated anti-rabbit IgG antibodies (cat. no. 2172707, Sigma-Aldrich, USA) at dilution 1:2000 for 40 min. The targeted protein expression was visualized by stained with Strep-FITC (Sigma-Aldrich, USA) at dilution 1:500. Nuclei were counterstained with 0.1 μg/mL 4′,6-diamidino-2-phenylindole (TOCRIS bioscience, USA). Protein expression and localization was detected using a fluorescent microscope with ApoTome system (Carl Zeiss, Germany).

### Statistical analysis

All experiments were repeated using cells derived from at least four different donors (n = 4). The statistical analysis was performed using Prism 9 for macOS (GraphPad Software, CA, USA). The Mann Whitney U test was used for two independent group comparisons. For three or more group comparison, significant differences were determined using the Kruskal Wallis test followed by pairwise comparison. Statistical significance was considered at *p* < 0.05.

## Supplementary Information

Supplementary Information.

## Data Availability

The datasets generated during and/or analysed during the current study are available from corresponding author upon reasonable request.
