# Supplementary material for: Non-canonical Wnt signaling participates in Jagged1-induced osteo/odontogenic differentiation in human dental pulp stem cells
Source: Sci Rep. 2022 May 9;12:7583. doi: 10.1038/s41598-022-11596-9 (PMC9085777; doi:10.1038/s41598-022-11596-9)
Supplement: Supplementary file 1 — Supplementary Information. [file 41598_2022_11596_MOESM1_ESM.docx]

**Non-canonical Wnt signaling participates in Jagged1-induced osteo/odontogenic differentiation in human dental pulp stem cells**

Chatvadee Kornsuthisopon^1,^ ^†^, Ajjima Chansaenroj^2, †^, Jeeranan Manokawinchoke^1^, Kevin A. Tompkins^3^, Nopadon Pirarat^2^*, Thanaphum Osathanon^1,4^*

^1^Dental Stem Cell Biology Research Unit, Faculty of Dentistry, Chulalongkorn University, Bangkok, 10330 Thailand

^2^Department of Pathology, Faculty of Veterinary Science, Chulalongkorn University, Bangkok, 10330 Thailand

^3^Office of Research Affairs, Faculty of Dentistry, Chulalongkorn University, Bangkok, 10330 Thailand

^4^Department of Anatomy, Faculty of Dentistry, Chulalongkorn University, Bangkok, 10330 Thailand

^†^ These authors contributed equally.

**Email:**

Chatvadee Kornsuthisopon: plearn.korn@gmail.com

Ajjima Chansaenroj: ajjimachansaenroj@gmail.com

Jeeranan Manokawinchoke: Jeeranan.M@chula.ac.th

Kevin A. Tompkins: kevin0318@yahoo.com

**Corresponding authors**

Thanaphum Osathanon, DDS, Ph.D.

Dental Stem Cell Biology Research Unit, Faculty of Dentistry,

Chulalongkorn University,

34 Henri-Dunant Rd. Pathumwan, Bangkok, 10330 Thailand

Tel: +66-2-218-8885, Fax: +66-2-218-8870

Email: [thanaphum.o@chula.ac.th](mailto:thanaphum.o@chula.ac.th)

Nopadon Pirarat, D.V.M., Ph.D.

Department of Pathology, Faculty of Veterinary Science,

Chulalongkorn University

39 Henri-Dunant Rd. Pathumwan, Bangkok, 10330 Thailand

Tel: +66-2-218-9612, Fax: +66-2-252-0779

E-mail: nopadonpirarat@gmail.com

**Supplementary Table 1.** Primer sequences

| **Gene** | **Primer sequences (F=forward, R=Reverse)** | **Accession number** |
| --- | --- | --- |
| *18S* | F: 5’-GGCGTCCCCCAACTTCTTA-3’ | NR003286.2 |
|  | R: 5’-GGGCATCACAGACCTGTTATT-3’ |  |
| *HES1* | F: 5’-AGGCGGACATTCTGGAAATG-3’ | NM_005524.2 |
|  | R: 5’-CGGTACTTCCCCAGCACACTT-3’ |  |
| *HEY1* | F: 5’-CTGCAGATGACCGTGGATCA-3’ | NM_012258.3 |
|  | R: 5’-CCAAACTCCGATAGTCCATAGCAA-3’ |  |
| *WNT2B* | F: 5’-TGGATGCCAAGGAGGAGGCT-3’ | NM_004185 |
|  | R: 5’-GTACAGGAACCACTCACGCCAT-3’ |  |
| *WNT5A* | F: 5’-TCAGGCACCATTAAACCACA-3’ | NM_003392.7 |
|  | R: 5’-AATTCACAGAGGTGTTGCAGC-3’ |  |
| *WNT5B* | F: 5’-CGTGGAGTACGGCTACCGCT-3’ | NM_032642 |
|  | R: 5’-CAGGCTACGTCTGCCATCTTAT-3’ |  |
| *WNT16* | F: 5’-AGTATGGCATGTGGTTCAGCA-3’ | NM_057168.2 |
|  | R: 5’-GCGGCAGTCTACTGACATCAA-3’ |  |
| *DKK1* | F: 5’-GCCTCAGGATTGTGTTGTGC-3’ | NM_012242.4 |
|  | R: 5’-ATCCGGCAAGACAGACCTTC-3’ |  |
| *DKK2* | F: 5’-AGTGTGAAGTTGGGAGGTATTGCC-3’ | NM_014421.2 |
|  | R: 5’-TGCCATTATTGCAGCGGGTACTG-3’ |  |
| *SOST* | F: 5’-ACTTCAGAGGAGGCAGAAATGG-3’ | NM_025237.2 |
|  | R: 5’-CAAGGGGGAATCTTATCCAACTTTC-3’ |  |
| *RUNX2* | F: 5’-ATGATGACACTGCCACCTCTGA-3’ | NM_001024630.3 |
|  | R: 5’-GGCTGGATAGTGCATTCGTG-3’ |  |
| *OSX* | F: 5’-GCCAGAAGCTGTGAAACCTC-3’ | NM_152860.1 |
|  | R: 5’-GCTGCAAGCTCTCCATAACC-3’ |  |
| *ALP* | F: 5’-CGAGATACAAGCACTCCCACTTC-3’ | NM_000478.3 |
|  | R: 5’-CTGTTCAGCTCGTACTGCATGTC-3’ |  |
| *COL1A1* | F: 5’-GTGCTAAAGGTGCCAATGGT-3’ | NM_000088.3 |
|  | R: 5’-ACCAGGTTCACCGCTGTTAC-3’ |  |
| *OCN* | F: 5’-CTTTGTGTCCAAGCAGGAGG-3’ | NM_199173.4 |
|  | R: 5’-CTGAAAGCCGATGTGGTCAG-3’ |  |
| *OPN* | F: 5’-AGGAGGAGGCAGAGCACA-3’ | NM_001040060.1 |
|  | R: 5’-CTGGTATGGCACAGGTGATG-3’ |  |
| *AXIN2* | F: 5’-ATGATTCCATGTCCATGACG-3’ | NM_001363813.1 |
|  | R: 5’-CTTCACACTGCGATGCATTT-3’ |  |
| *CaMKII* | F: 5’-TCAAGCCCCAGACAAACAG-3’ | NM_172079.3 |
|  | R: 5’-TTCCTTAATGCCGTCCACTG-3’ |  |
| *ROR2* | F: 5’-GAAGAGGACGACGACGAGGT-3’ | NM_001318204.2 |
|  | R: 5’-CGGGACACTGAGAGCAGAAG-3’ |  |


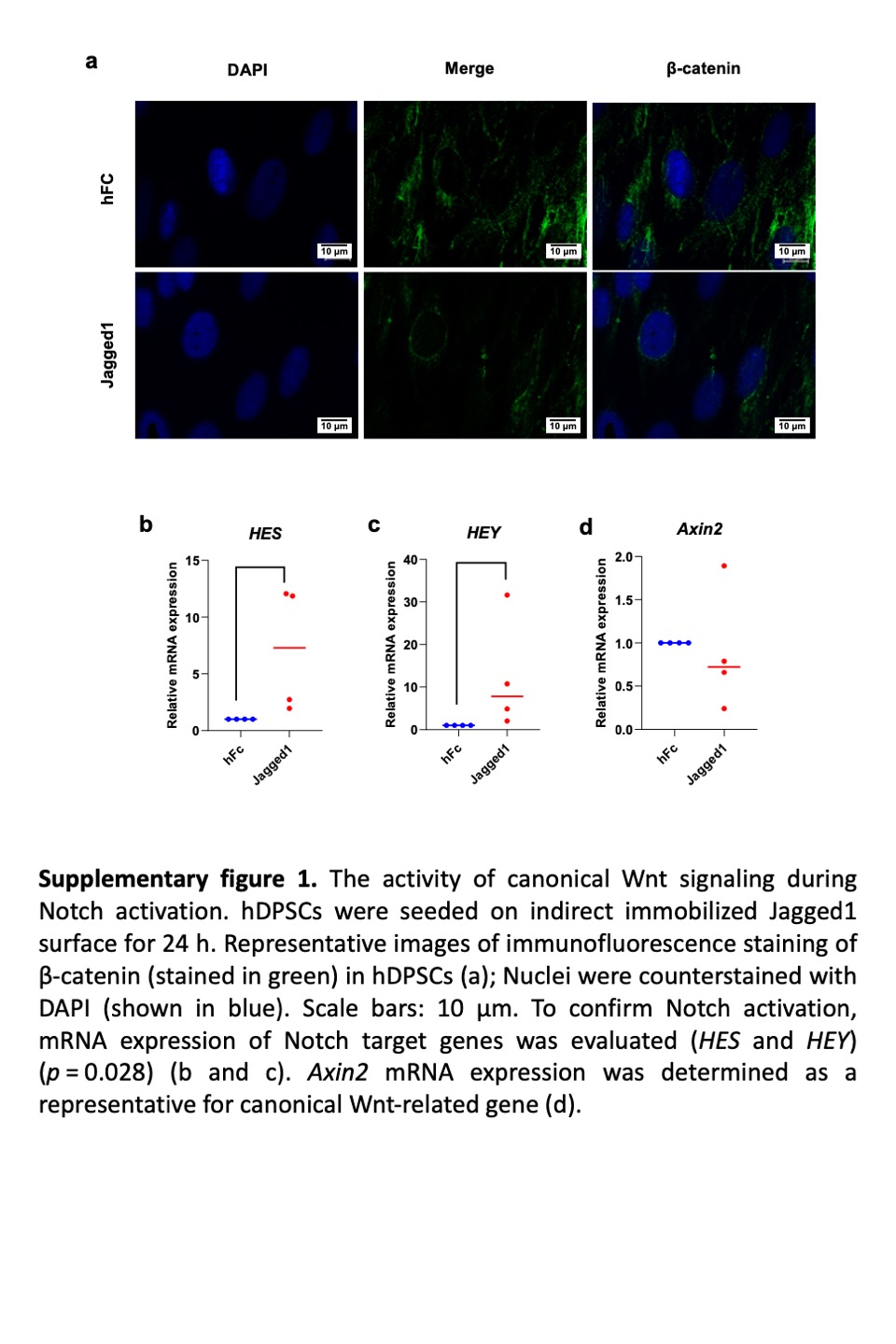


**Supplementary figure 1.** The activity of canonical Wnt signaling during Notch activation. hDPSCs were seeded on an indirect immobilized Jagged1 surface for 24 h. Representative images of immunofluorescence staining of β-catenin (stained in green) in hDPSCs (a); Nuclei were counterstained with DAPI (shown in blue). Scale bars: 10 µm. To confirm Notch activation, mRNA expression of Notch target genes was evaluated (*HES* and *HEY*) (*p* = 0.028) (b and c). *Axin2* mRNA expression was determined as a representative for canonical Wnt-related gene (d).

**
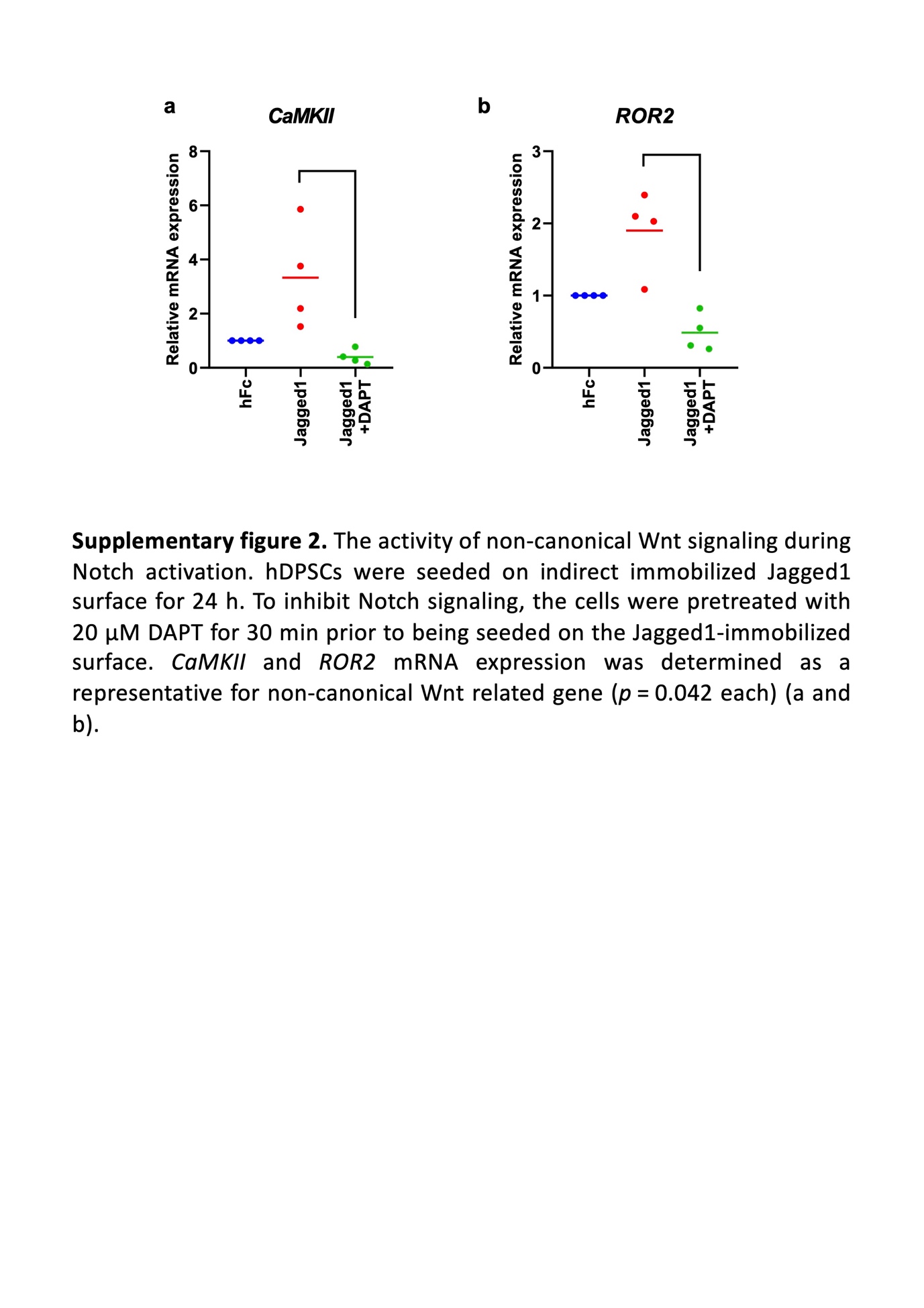
**

**Supplementary figure 2.** The activity of non-canonical Wnt signaling during Notch activation. hDPSCs were seeded on an indirect immobilized Jagged1 surface for 24 h. To inhibit Notch signaling, the cells were pretreated with 20 μM DAPT for 30 min before being seeded on the Jagged1-immobilized surface. *CaMKII* and *ROR2* mRNA expression was determined as representative for non-canonical Wnt-related genes (*p* = 0.042 each) (a and b).
